# Development of an ELISA for sensitive and specific detection of IgA autoantibodies against BP180 in pemphigoid diseases

**DOI:** 10.1186/1750-1172-6-31

**Published:** 2011-05-28

**Authors:** Kinga Csorba, Sabine Schmidt, Florina Florea, Norito Ishii, Takashi Hashimoto, Michael Hertl, Sarolta Kárpáti, Leena Bruckner-Tuderman, Wataru Nishie, Cassian Sitaru

**Affiliations:** 1 Department of Dermatology, University of Freiburg, Hauptstrasse 7, 79104, Freiburg, Germany; 2 Faculty of Biology, University of Freiburg, Schaenzlestrasse 1, 79104, Freiburg, Germany; 3 Department of Dermatology, Kurume University, 67 Asahimachi, Kurume, Fukuoka 830-0011, Japan; 4 Department of Dermatology and Allergology, University of Marburg, Deutschhausstrasse 9, 35037, Marburg, Germany; 5 Department of Dermatology, Venerology and Dermatooncology, Faculty of Medicine, Semmelweis University, Maria street 41, 1085, Budapest, Hungary; 6 BIOSS Center for Biological Signaling Studies, University of Freiburg, Hebelstrasse 25, 79104, Freiburg, Germany

## Abstract

**Background:**

Pemphigoids are rare diseases associated with IgG, IgE and IgA autoantibodies against collagen XVII/BP180. An entity of the pemphigoid group is the lamina lucida-type of linear IgA disease (IgA pemphigoid) characterized by IgA autoantibodies against BP180. While for the detection of IgG and IgE autoantibodies specific to collagen XVII several ELISA systems have been established, no quantitative immunoassay has been yet developed for IgA autoantibodies. Therefore, the aim of the present study was to develop an ELISA to detect IgA autoantibodies against collagen XVII in the sera of patients with pemphigoids.

**Methods:**

We expressed a soluble recombinant form of the collagen XVII ectodomain in mammalian cells. Reactivity of IgA autoantibodies from patients with IgA pemphigoid was assessed by immunofluorescence microscopy and immunoblot analysis. ELISA test conditions were determined by chessboard titration experiments. The sensitivity, specificity and the cut-off were determined by receiver-operating characteristics analysis.

**Results:**

The optimized assay was carried out using sera from patients with IgA pemphigoid (n = 30) and healthy donors (n = 105). By receiver operating characteristics (ROC) analysis, an area under the curve of 0.993 was calculated, indicating an excellent discriminatory capacity. Thus, a sensitivity and specificity of 83.3% and 100%, respectively, was determined for a cut-off point of 0.48. As additional control groups, sera from patients with bullous pemphigoid (n = 31) and dermatitis herpetiformis (n = 50), a disease associated with IgA autoantibodies against epidermal transglutaminase, were tested. In 26% of bullous pemphigoid patients, IgA autoantibodies recognized the ectodomain of collagen XVII. One of 50 (2%) of dermatitis herpetiformis patients sera slightly topped the cut-off value.

**Conclusions:**

We developed the first ELISA for the specific and sensitive detection of serum IgA autoantibodies specific to collagen XVII in patients with pemphigoids. This immunoassay should prove a useful tool for clinical and translational research and should essentially improve the diagnosis and disease monitoring of patients with IgA pemphigoid. Moreover, our findings strongly suggest that IgA pemphigoid and IgG bullous pemphigoid represent two ends of the clinical spectrum of an immunological loss of tolerance against components of hemidesmosomes, which is mediated by both IgG and IgA autoantibodies.

## Background

**Figure 1:**
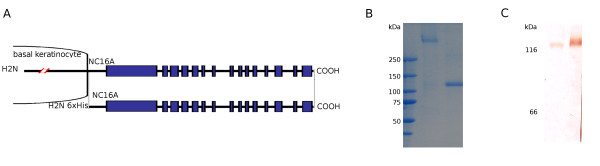
**Recombinant ectodomain of BP180 used in this study**. A: Schematic representation of human BP180 and its ectodomain consisting of alternating collagenous (C) and non-collagenous (NC) domains. The recombinant form of BP180 ectodomain used for the development of the current ELISA system spans from aa 490 through aa 1497 and has an NH_2_-terminal hexahistidine tag. B: Sodium dodecyl sulfate-polyacrylamide gel electrophoresis of the purified recombinant protein without and with previous boiling, which migrates around 360 (lane 2) and 120 kDa (lane 3), respectively. C: Immunoblot analysis of the native, normal human keratinocyte-derived shed BP180 ectodomain (lane 1) and the recombinant BP180 ectodomain (lane 2) using a monoclonal antibody specific to BP180. Weight markers of 116 and 66 kDa are shown to the left.

Pemphigoids are rare autoimmune blistering disorders associated with autoimmunity against hemidesmosomal proteins [1]. Main entities of the pemphigoid group include bullous pemphigoid, pemphigoid gestationis, linear IgA disease, mucous membrane pemphigoid and lichen planus pemphigoides with an approximate annual incidence of 7, 0.5, 0.5, 1 and undefined cases in one million, respectively [2-5]. A major target of pemphigoid autoantibodies is the bullous pemphigoid antigen of 180 kDa (BP180), also referred to as collagen XVII, a hemidesmosomal transmembrane protein with a type II orientation whose extracellular domain consists of 15 collagenous regions interrupted by non-collagenous portions (Figure 1A) [1,4,6]. In a minority of patients, IgA reactivity against BP230, an intracellular hemidesmosomal component, has been detected [7]. A hallmark of collagen XVII is its constitutive shedding yielding a shorter and soluble form of the molecule that spans most of its ectodomain [8,9].

BP180 is targeted by autoantibodies of different Ig isotypes, including different IgG subclasses, IgA and IgE [10-13]. The pathogenic relevance of IgG autoantibodies against BP180 is supported by several lines of evidence: 1) the transplacental transfer of pemphigoid IgG autoantibodies from mothers to the fetus induces transient skin blistering in the newborn [14-16]; 2) serum levels of IgG autoantibodies against BP180 correlate with disease activity in patients with bullous pemphigoid and pemphigoid gestationis [17-20]; 3) patients autoantibodies against BP180 recruit leukocytes to the dermal-epidermal junction and induce dermal-epidermal separation of human skin [21,22]; 4) IgG antibodies against BP180 induce subepidermal blistering when passively transferred into neonatal autoantigen humanized, wild type mice and hamsters [23-26]; 5) grafting of human BP180 transgenic mouse skin induces an autoimmune response resulting in subepidermal blistering in wild-type animals [27,28]. IgE autoantibodies against BP180 correlate with disease activity in pemphigoid patients and induce eosinophil infiltration and dermal-epidermal separation when injected into human skin grafted on immunodeficient mice [29-31].

While the pathogenic potential of IgG and IgE autoantibodies against BP180 was characterized *ex vivo *and in animal models, the pathogenicity of IgA autoantibodies was relatively less studied [32]. Very recently, we demonstrated that IgA autoantibodies from patients with linear IgA disease induce granulocyte-dependent dermal-epidermal separation in cryosections of human skin (van der Steen et al, unpublished).

Linear IgA disease was defined as a new entity different from dermatitis herpetiformis on the basis of a linear IgA deposition at the dermal-epidermal junction [33,34]. Further studies revealed heterogeneous molecular specificity of the IgA autoantibodies in patients with linear IgA disease, including BP180, BP230, collagen VII as well as still unidentified antigens of 180-, 200-, and 285-kDa [35]. While in most patients, IgA autoantibodies bind to the epidermal side of the salt-split skin by indirect immunofluorescence (IF) microscopy, staining of the dermal side of the artificial split may be also detected. IgA autoantibodies from pemphigoid patient sera recognize several forms of BP180 and preferentially bind to proteolytic products of this autoantigen [36-38]. Initial studies have shown that a 97 kDa protein (LABD97) is responsible for basement membrane binding of IgA in patients with linear IgA disease and that this protein is recognized in human skin extracts by IgA autoantibodies, when immunoblot analysis is performed [36]. Further studies have shown that a 120 kDa protein, referred to as linear IgA disease antigen (LAD)-1, secreted into the supernatants of cultured keratinocytes is also target of linear IgA disease autoantibodies [37]. Based on antigenic cross-reactivity between LAD-1 and full-length BP180 it has been postulated that LABD97 and LAD-1 are fragments of BP180 [38]. Subsequent independent studies confirmed that LABD97 and LAD-1 indeed represent portions of the extracellular domain of BP180 [8,9,39].

For the detection of autoantibodies against BP180, belonging to different immunoglobulin isotypes, several ELISA systems have been established. To detect IgG class autoantibodies from patients' sera has been the focus of immunoassays developed until now [19,20,40-43]. Two of these test systems are commercially available and widely used in diagnosis. Recently, ELISA systems using a recombinant form of BP180 NC16A domain were established for the detection of specific IgE in pemphigoid patients [31,44]. In contrast, an ELISA for the detection of IgA autoantibodies in pemphigoid diseases has not yet been developed. Both, (i) IgA deposition at the dermal-epidermal junction being a major diagnostic criterion and (ii) the demonstration of the pathogenic potential of IgA autoantibodies in patients with linear IgA disease emphasize the need of elaborating a sensitive and specific immunoassay to detect the IgA autoantibodies in this orphan disease.

Therefore, in the present work, we used a recombinant form of the ectodomain of human BP180 to establish an ELISA for the detection of IgA autoantibodies. Our results show that ELISA using the ectodomain of BP180 is a sensitive and most specific system for detection of circulating IgA autoantibodies in patients with pemphigoids.

## Materials and methods

### Human sera

Serum samples were obtained from patients with linear IgA disease (n = 30, mean age 56 years), bullous pemphigoid (n = 31, mean age 76.5 years), and dermatitis herpetiformis (n = 50, mean age 43 years) before initiation of treatment, as well as from healthy donors (n = 105, mean age 43 years). Our present study focuses on the IgA pemphigoid subgroup of the linear IgA disease patients, characterized by IgA binding to the epidermal side of the salt-split skin by indirect IF microscopy. Sera from patients with IgA epidermolysis bullosa acquisita or from patients with laminin autoimmunity were not included. The clinical diagnosis of IgA pemphigoid was confirmed by (i) subepidermal blisters, (ii) linear IgA deposition along the dermal-epidermal junction of perilesional skin by direct IF microscopy, and (iii) circulating IgA autoantibodies binding to the epidermal side of 1M salt-split skin by indirect IF microscopy. The clinical diagnosis of bullous pemphigoid was validated by (i) subepidermal blisters with inflammatory infiltrate, (iii) linear IgG deposition at the basement membrane zone revealed by direct IF microscopy, and (iii) circulating IgG autoantibodies to the epidermal side of 1M salt-split skin as shown by indirect IF microscopy and ELISA using a recombinant form of the 16^th ^non-collagenous region of BP180 expressed as a glutathione-S-transferase fusion protein. Patients with both IgG and IgA deposition at the dermal-epidermal junction were diagnosed as bullous pemphigoid. For the experiments conducted, we obtained approval from the Ethics Committee of the Medical Faculty of the University of Freiburg, Germany (Institutional Board Projects no 318/07 and 407/08). We obtained informed consent from patients whose material was used in the study, in adherence to the Helsinki Principles.

### Cell culture

HaCaT human keratinocytes were cultured in serum free, low calcium KGM medium, supplemented with KGM Supplement Mix and CaCl_2 _(all from PromoCell), as well as L-glutamine, penicillin and streptomycin (all from Biochrome). Transfected Flp-In HEK 293T cells (Flp-In™-293, Invitrogen) were cultured in DMEM medium, without phenol red (Gibco) supplemented with 10% FCS, L-glutamine, penicillin, streptomycin and hygromycin (all from Biochrome).

### Production of recombinant BP180 ectodomain

The ectodomain of BP180, corresponding to amino acids 490-1497 was produced by stable expression in Flp-In HEK-293 cells (Invitrogen) as reported [45]. Briefly, the recombinant protein encoding for the ectodomain of human BP180 with an N-terminal Ig κ chain signal sequence and hexahistidine tag was affinity purified from culture medium by metallochelate affinity using Ni-NTA (Qiagen). Alternatively, the recombinant protein from harvested culture media was precipitated with 45% ammonium sulphate followed by dialysis against PBS. The purified protein's concentration was determined by spectrophotometry at 280 nm and this protein was subsequently used to determine the concentration of the specific protein in the precipitated protein by densitometric comparison of the specific bands of the Coomassie stained gel using the ImageJ software (http://rsb.info.nih.gov/ij).

### Enzyme-linked immunosorbent assays

IgG pemphigoid autoantibodies were detected by ELISA utilizing a recombinant form of the 16^th ^non-collagenous region of BP180 expressed as a glutathione-S-transferase fusion protein following manufacturer's instructions (MBL Co, Nagoya, Japan). For detection of IgA autoantibodies, 96-well microtiter plates (Greiner Bio-One, Germany) were coated with 1.4 μg/well of recombinant BP180 ectodomain in 0.1 M bicarbonate buffer (pH 9.6), overnight at 4 °C. After washing with 0.05%Tween20-PBS (w/v) and subsequent 1 h blocking with 2% BSA-PBS (w/v) the plates were incubated for 1 h with 1:50 diluted sera in 1% BSA-0.05% Tween20-PBS (w/v). Bound antibodies were detected using a 1000-fold dilution of a horseradish-peroxidase (HRP) conjugated rabbit anti-human IgA antibody (ab8510, Abcam) and orthophenylene diamine (Dako). All steps were carried out at room temperature. The optical density (OD) was read at 490 nm using an automated spectrophotometer (Sirius HT-TRF, MWG). Each serum was tested in duplicate. The cut-off for positivity was validated and optimized by receiver-operating characteristics (ROC) analysis as described below and was defined as 0.48 OD units at 490 nm.

In order to rule out the possibility of a nonspecific cross-reaction between the secondary HRP-labeled anti human IgA antibody and the IgG autoantibodies in bullous pemphigoid patients' sera we performed the ELISA described above with sera of known high IgA and IgG titers from IgA pemphigoid and bullous pemphigoid patients, respectively. Normal human serum was used as control. Bound IgG and IgA antibodies were detected in all samples with a 5000-fold diluted, HRP-labeled goat anti-human IgG antibody (ab6858, Abcam) and the HRP conjugated anti-human IgA antibody.

IgG autoantibodies against BP230 were detected by the MESACUP BP230 commercial ELISA kit (MBL Co, Nagoya, Japan) following the manufacturer's instructions [46]. For the detection of IgA autoantibodies against BP230, we have slightly modified the kit protocol. Briefly, BP230 microwell strips were incubated with 50 times diluted linear IgA disease patients' sera and appropriate control sera. After washing, the bound antibodies were detected using 1000-fold diluted HRP-conjugated anti-human IgA antibody (ab8510, Abcam) and the tetramethylbenzidine dihydrochloride (TMB)/ hydrogen peroxide substrate solution from the kit.

### SDS-PAGE and immunoblot analysis

Immunoblotting with recombinant or native BP180 ectodomain proteins was performed as described with minor modification [18,47]. Briefly, preparations of recombinant BP180 ectodomain, native shed BP180 and precipitated supernatant of empty pcDNA5/FRT transfected HEK-293 cells were separated by SDS-PAGE on 6-8% preparative gels, under reducing conditions, followed by transfer onto nitrocellulose (Whatman/Protran BA85). Membrane strips were incubated overnight with 50-fold diluted serum or 800-fold diluted mouse mAb NC16A-3 directed against the 16^th ^non-collagenous domain of BP180. Reactivity was visualized with secondary, HRP-conjugated rabbit anti-human IgA (Abcam) or anti-mouse IgG (BioRad) antibodies and diaminobenzidine (Merck).

### Indirect immunofluorescence

Detection of circulating autoantibodies by indirect immunofluorescence followed published protocols [18,47]. Briefly, frozen sections of salt-split normal human skin were incubated in a first step with 10-fold diluted sera from patients with IgA pemphigoid, bullous pemphigoid and healthy donors and in a second step with 40-fold diluted, fluorescein isothiocyanate (FITC) conjugated polyclonal goat anti-human IgA antibody (Invitrogen).

### Statistical analysis

Diagnostic parameters of our new ELISA were optimized by using the ROC analysis. Therefore, to determine the cut-off value for the ELISA using recombinant BP180 ectodomain, we performed a ROC analysis by plotting on the X-axis the 1 - specificity (the false positive rate) and on the Y-axis the sensitivity (the true positive rate). The diagnostic sensitivity and specificity are a function of the selected cut-off value. In the diagnostic context of pemphigoid diseases, which have a very low prevalence, it is advisable to choose a cut-off to maximise the specificity of the assay. In this regard, a specificity of 97.5% or higher may be considered an optimal target. A similar specificity would be aimed by applying an alternative method of calculating the cut-off based on the mean plus two standard deviations (2SD) of the negative reference sample [48]. Correlations were analyzed by the Spearman's rank correlation test. Proportion comparison was examined by Fisher's exact test. Data are considered significantly different if the p <0.05. Statistical analyses were performed using the GraphPad Prism statistical package (v5; GraphPad Software, San Diego, CA).

## Results

### Generation of the recombinant autoantigen

When separated by SDS-PAGE without or with previous boiling, the recombinant protein migrated consistently with the calculated molecular masses of 360 kDa (Figure 1B, lane 2) and 120 kDa (Figure 1B, lane 3), for its trimeric and monomeric forms, respectively. This SDS-PAGE migration pattern matched the one observed for the keratinocyte-derived shed ectodomain of BP180 protein (data not shown) indicating that the recombinant protein forms a native-like collagenous trimerized structure. Monoclonal antibodies specific for the BP180NC16A domain of BP180, recognized the keratinocyte-derived shed ectodomain (Figure 1C, lane 1) and the recombinant protein by immunoblot analysis (Figure 1C, lane 2).

### Immunoreactivity of recombinant BP180 with IgA autoantibodies

**Figure 2:**
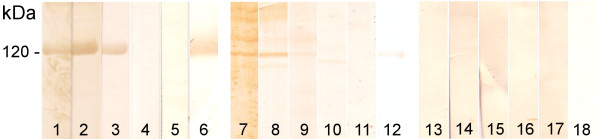
**Immunoreactivity of IgA autoantibodies with native and recombinant BP180 ectodomain**. Precipitated, recombinant (lanes 1-6) and native, keratinocyte-derived (lanes 7-12) BP180 ectodomain immunoblotted with IgA pemphigoid (LAD) patients' sera (lanes 1-3, 7-9) and control sera (NHS) (lanes 4, 5, 10 and 11). As substrate control (lanes 13-18), precipitated culture medium from cells transfected with empty vector was immunoblotted using the same LAD (lanes 13-15) and NHS (lane 16, 17) sera. Presence or absence of the 120 kDa ectodomain was visualized using a specific monoclonal Ab (lanes 6, 12 and 18).

IgA autoantibodies from reference IgA pemphigoid patients' sera (n = 5) recognized the recombinant and the native BP180 ectodomain by immunoblotting. None of the normal human sera reacted with the autoantigens and IgA antibodies from none of the tested sera bound to the precipitated proteins of the empty vector transfected HEK cell culture medium. Representative examples are shown in Figure 2.

### Development of ELISA using recombinant BP180

**Figure 3:**
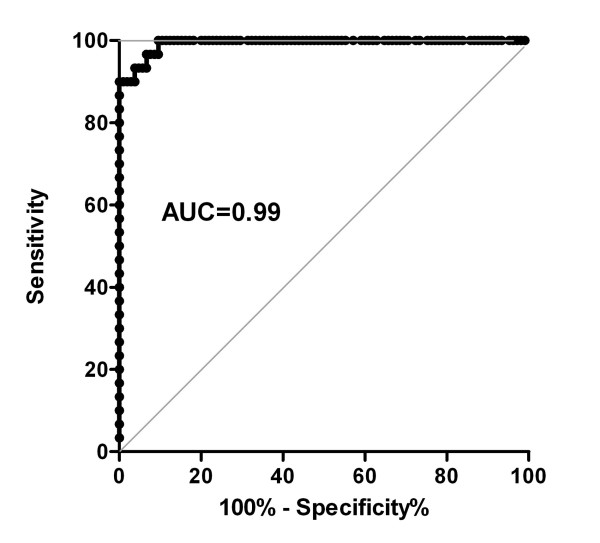
**Receiver-operating-characteristic (ROC) curve**. AUC, area under the curve. Test performed with sera from IgA pemphigoid patients (n = 30) and controls (n = 105).

The working conditions, including antigen amount/well, dilution of sera and secondary antibodies have been defined by an initial chessboard titration (data not shown). The secondary HRP-labeled anti-human IgA antibody was tested by ELISA (see Methods) in order to rule out nonspecific cross-reactivity with IgG autoantibodies. No cross-reactivity was found (data not shown). To determine the cut-off value of the newly established immunoassay, we performed a ROC analysis of the ELISA readings with sera from 30 IgA pemphigoid patients and 105 healthy donors as controls. Based on a calculated specificity of 100% and a sensitivity of 83.3% the cut-off was set at 0.48 OD reading units (Figure 3).

### ELISA using recombinant BP180 is a sensitive and specific tool to detect IgA autoantibodies in pemphigoid patients

**Figure 4:**
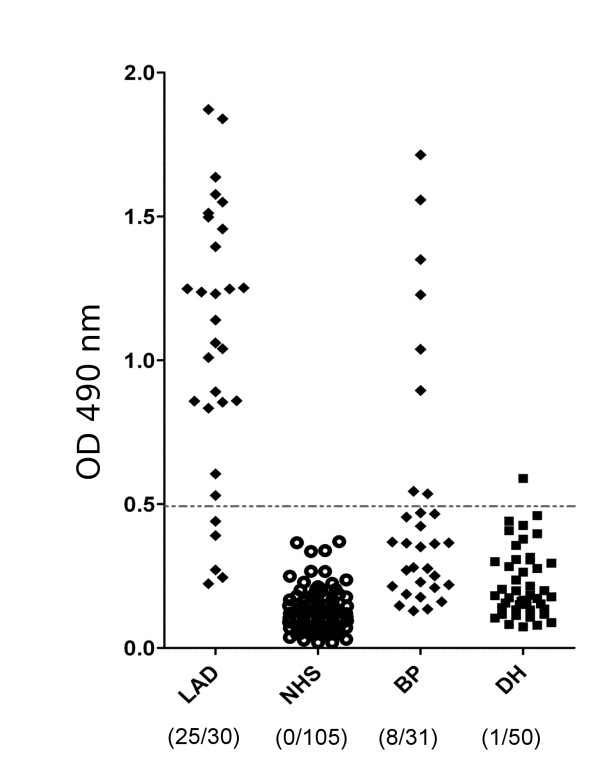
**ELISA reactivity of linear IgA disease and control sera with the recombinant BP180 ectodomain**. Scatter plots represent corrected optical density measurements of serum reactivity of IgA pemphigoid (LAD), dermatitis herpetiformis (DH), bullous pemphigoid (BP) patients and age matched, healthy donors (NHS) with the recombinant BP180 ectodomain_. _The cut-off of the assay is represented by a dashed line. Numbers in brackets are positive and total cases, respectively.

Applying the cut-off value of 0.48 defined by ROC analysis for the newly developed ELISA showed that 25 (83.3%; 95% CI: 65.2%-94.3%) of IgA pemphigoid and 8 (26.0%; 95% CI: 12.0%-45.0%) of bullous pemphigoid patients were positive, while 1 (2%; 95% CI: 0.05%-10.0%) of patients with dermatitis herpetiformis slightly topped the cut-off value (Figure 4). Serum from none of the healthy donors (0%; 95% CI: 0.0%-4.0%) showed IgA reactivity with the recombinant BP180 ectodomain (Table 1). Therefore, a sensitivity and a specificity of 83.3% (95% CI: 70-97%) and 100% (95% CI: 96-100%), respectively, were calculated for the ELISA detecting IgA autoantibodies against BP180 in patients with IgA pemphigoid. The area under the curve (AUC) was 0.993 (95% CI.: 98.4%-100%). IgA autoantibodies against BP180 ectodomain were primarily detected in bullous pemphigoid patients showing IgG reactivity against the BP180 NC16A region by ELISA (n = 8). IgA autoantibodies against BP180 ectodomain were found in only 1 of 16 bullous pemphigoid (6.25%, 95% CI.: 0%-28%) patients with negative BP180 NC16A.

**Table 1:** Sensitivity and specificity of the IgA ELISA with recombinant BP180 ectodomain

Sera	Positive/Total	Sensitivity (95% CI)	Specificity (95%CI)
LAD	25/30 (83%)	83.3% (65.2%-94.3%)	100% (96.55%-100%)
BP	8/31 (26%)	26% (12.0%-45.0%)	100% (96.55%-100%)
DH	1/50 (2%)	2% (0.05%-10.0%)	100% (96.55%-100%)
NHS	0/105 (0%)	0% (0.0%-4.0%)	100% (96.55%-100%)

### Levels of BP230-specific IgA and IgG autoantibody in IgA pemphigoid patients' sera

While BP180 is considered the major autoantigen in IgA pemphigoid, BP230 has been also documented as target of these autoantibodies. Therefore, we have measured the IgA and IgG levels agains BP230 by ELISA in our 30 IgA pemphigoid patients' sera. In 5 patients, we have detected IgA autoantibodies against BP230, whereas none of the sera had detectable IgG against BP230 (Table 2).

**Table 2:** Immunoreactivity profile of linear IgA disease patients

	Positive indirect immunofluorescence	Immunoblot with native shed BP180 ectodomain		Immunoblot with recombinant BP180 ectodomain		ELISA BP230-IgA		ELISA BP230-IgG	
		
		*positive*	*negative*	*positive*	*negative*	*positive*	*negative*	*positive*	*negative*
ELISA BP180 positive	25 (83%)	20(77%)	3 (11.5%)	20 (71.4%)	4 (14.3%)	4 (13.3%)	21 (70%)	0 (0%)	25 (83%)
ELISA BP180 negative	5 (17%)	1 (3.8%)	2 (7.7%)	1 (3.6%)	3 (10.7%)	1 (3.3%)	4 (13.3%)	0 (0%)	5 (17%)
	n = 30		n = 26		n = 28		n = 30		n = 30

n-total number of tested sera

### IgA levels by BP180 ELISA correlate with the IgA reactivity against the dermal-epidermal junction by IF microscopy

**Figure 5:**
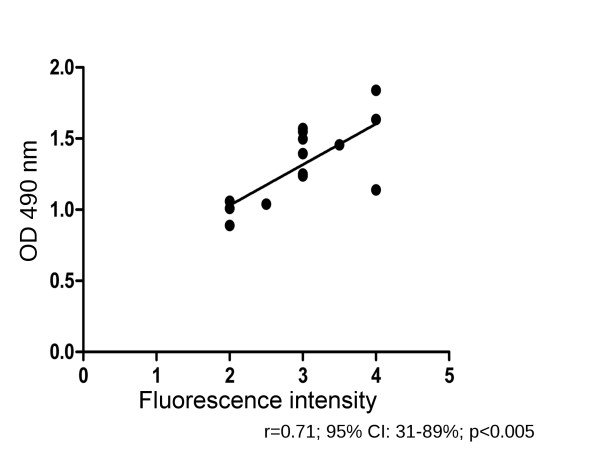
**Correlation of BP180-specific IgA levels by ELISA with IgA reactivity by IF microscopy in IgA pemphigoid patients**. Spearman rank coefficient (r) = 0.71, 95% confidence interval 31-89%, p < 0.005, n = 16.

The indirect IF microscopy on salt-split skin is a standard diagnostic tool in pemphigoid diseases. To further characterize the suitability of the newly developed ELISA for diagnosis of pemphigoid diseases, we correlated in IgA pemphigoid patients the IgA levels by ELISA with the semi-quantitative reactivity scores by IF microscopy on salt-split skin. When the IgA levels of BP180-specific IgA autoantibodies were plotted against the IgA indirect IF microscopy scores, a positive correlation (r = 0.71; 95% CI: 31-89%; p < 0.005) was obtained (Figure 5).

### Analysis of IgA immunoreactivity against BP180 ectodomain in bullous pemphigoid patients

The reactivity of IgA autoantibodies from sera of bullous pemphigoid patients was analyzed by IF microscopy using frozen sections of human salt-split skin. Representative examples are shown in Additional file 1. Thirteen of 31 bullous pemphigoid sera had detectable autoreactive IgA antibodies binding to the epidermal side of the split skin. When immunoblot analysis was performed to identify IgA autoreactivity, we found that 13 and 19 of 31 patients recognized the native and the recombinant BP180 ectodomain, respectively. Characteristic examples are depicted in Additional file 1.

### Comparison of different immunoassays for the detection of BP180-specific IgA autoantibodies in pemphigoid diseases

We compared using Fisher's exact test the findings in IgA pemphigoid patients obtained by ELISA using recombinant BP180 ectodomain and immunoblotting with native and recombinant LAD-1 antigen. Results are summarized in Table 2. The calculated p values of 0.703 and 0.502, respectively, show that the ELISA and immunoblotting results are not significantly different. Discrepancies were observed in only 4 (15%) and 5 (18%) patients having different results by ELISA compared with immunoblotting with the native LAD-1 and ELISA compared with immunoblotting with recombinant BP180 ectodomain, respectively.

## Discussion

Several quantitative immunoassays for the detection of IgG and IgE autoantibodies against BP180 have already been established [19,20,31,40-44]. Using these tests, a plethora of clinical and experimental data have been generated, which decisively influenced our knowledge on disease pathogenesis and are firmly implemented in the diagnosis and monitoring of pemphigoid diseases [1,49]. In addition to IgG and IgE, IgA autoantibodies are present to different extent in pemphigoid diseases. While pemphigoid patients showing a predominant IgA autoimmune response directed against BP180 are diagnosed as linear IgA disease, IgA autoantibodies may complement a dominant IgG response in bullous pemphigoid or mucous membrane pemphigoid [1]. However, quantitative assays for measuring the levels of serum IgA autoantibodies in pemphigoid diseases have not been established yet. Therefore, in the present study, we developed an ELISA using recombinant BP180 for the detection of IgA autoantibodies.

For detecting the IgA autoantibodies in pemphigoid diseases we have used a recently generated recombinant form of the ectodomain of BP180 [45]. The ectodomain of the autoantigen was expressed in a human cell line to ensure optimal posttranslational modifications of the protein, which were shown to influence the binding of pemphigoid autoantibodies of the IgG class [50]. IgA reactivity was found by ELISA using a recombinant form of the 16^th ^non-collagenous region of BP180, which was expressed as a glutathione-S-transferase fusion protein in bacteria, in about 20% of IgA pemphigoid patients [51]. The expression of the antigen in mammalian cells and the fact that our recombinant form of BP180 contains its entire ectodomain significantly raised the sensitivity and specificity of the immunoassay and strongly support its use for the detection of IgA autoantibodies for the diagnosis of pemphigoid diseases.

The ELISA system developed in the present study was shown to be highly sensitive and specific for the detection of IgA autoantibodies in IgA pemphigoid. Since IgA reactivity with the epidermal side of the salt-split human skin by indirect IF microscopy was an inclusion criterion of the study patients, a direct comparison of the sensitivity of these 2 assays was not possible in the present work. The observation that IgA autoantibodies against BP180 were detected in bullous pemphigoid patients is in line with previous findings [7,52]. This intriguing finding prompted us to characterize the detection of IgA autoantibodies against BP180 by our ELISA in patients negative for IgG autoantibodies by the commercially available ELISA using the recombinant 16^th ^non-collagenous region of BP180 (MBL Co, Nagoya, Japan). However, only 6.25% of patients negative for IgG BP180NC16A ELISA showed IgA reactivity against the BP180 ectodomain. Nevertheless, our finding, that a significant proportion of the bullous pemphigoid patients show IgA autoantibodies against the ectodomain of BP180 suggests that the newly developed IgA ELISA may be a useful ancillary diagnostic tool in patients with bullous pemphigoid. In our patient cohort, only a minority of sera (approximately 16%) showed IgA reactivity against BP230, confirming that BP180 is the major autoantigen in IgA pemphigoid.

In addition to facilitating translational research focusing on the IgA autoimmune response in pemphigoid diseases, the newly developed ELISA could complement or replace the traditional semi-quantitative, observer-dependent and time-consuming IF microscopy on salt-split skin and immunoblotting using concentrated conditioned supernatant of cultured keratinocytes. In contrast to IgG reactivity [20], the ELISA levels of BP180-specific IgA correlated well with the IgA reactivity by indirect IF microscopy. In a recent study, we have investigated the potential of neoepitope-specific rabbit IgG antibodies to induce dermal-epidermal separation in an *ex vivo *assay [45]. Our results showed as proof-of-principle that neoepitope-specific antibodies are pathogenic. However, the pathogenic potential of neoepitope-specific patient IgA autoantibodies has not been directly addressed yet. Using the newly developed ELISA with further recombinant forms of the BP180 ectodomain, which may better reproduce the neoepitopes of the native shed ectodomain, may facilitate addressing the diagnostic relevance of measuring specifically the neoepitope-specific IgA autoantibodies.

In addition to healthy blood donors, our control group included patients with dermatitis herpetiformis. Dermatitis herpetiformis is a polymorphic autoimmune blistering disease with granular IgA and epidermal transglutaminase deposition in the papillary dermis, without circulating IgA autoantibodies binding to the basement membrane or hemidesmosomes. The disease is associated with a latent gluten-free enteropathy and the majority of patients have IgA type epidermal and tissue transglutaminase antibodies [53,54]. These diseases are associated with IgA autoantibodies staining the endomysium by IF microscopy and recognizing the tissue and epidermal transglutaminase [55,56]. Dermatitis herpetiformis shows overlapping clinical and histopathological features with the pemphigoids [1,53]. In 1979, linear IgA disease was defined as a new entity different from dermatitis herpetiformis on the basis of a linear IgA deposition at the dermal-epidermal junction, which still constitutes its golden diagnostic standard [33,34]. The characteristic diagnostic IF microscopy tests in dermatitis herpetiformis and linear IgA disease were complemented by the development of immunoassays for detecting IgA autoantibodies specific for tissue and epidermal transglutaminase [55-58]. The ELISA system developed in this study adds a new relevant tool, which further facilitates the positive and differential diagnosis of linear IgA disease and dermatitis herpetiformis.

In summary, our results establish an ELISA system for measuring IgA autoantibodies against BP180 in pemphigoid diseases. The newly developed ELISA is the first molecular quantitative immunoassay important for the diagnosis of linear IgA disease. In addition, this immunoassay should prove a useful tool for clinical research and could optimize the diagnosis and monitoring of pemphigoid diseases. Moreover, our findings strongly suggest that linear IgA disease and bullous pemphigoid represent two poles of the clinical spectrum of an immunological loss of tolerance against defined adhesion proteins of hemidesmosomes which is associated, with both IgG and IgA autoantibodies.

## Abbreviations

LAD: linear IgA disease; BP: bullous pemphigoid; DH: dermatitis herpetiformis; NHS: normal human sera.

## Competing interests

The authors declare that they have no competing interests.

## Authors' contributions

KC and CS designed and performed the ELISA, coordinated the data acquisition, analysed and interpreted the data and drafted the manuscript. SS produced the recombinant BP180 ectodomain and characterized its immunoreactivity. FF performed IF microscopy and immunoblot analysis of the major part of the sera in this study. WN provided us the transfected Flp-in HEK-293 cell line and made contributions to the conception of the study. NI, TH, MH, SK, and LBT provided serum samples used in the study and have participated in the experimental design and drafting of manuscript. All authors read and approved the final manuscript.

## Supplementary Material

Additional file 1 **Characterization of IgA autoreactivity in bullous pemphigoid (BP) patients**. A-F: Cryosections of human salt split skin were incubated with BP sera (A, B, D, E), serum from a patient with IgA pemphigoid (C), and a healthy donor (F). IgA autoantibodies were detected using a FITC-labeled goat anti-human IgA antibody (magnification, 200x). G: Keratinocyte-derived shed (lanes 1-5) and recombinant BP180 ectodomain (lanes 6-10) were separated by 6% SDS-PAGE and electrophoretically transferred to nitrocellulose. The membranes were immunoblotted with serum from BP patients (lanes 3-5 and 8-10), a healthy donor (lane 2 and 7) and a BP180-specific mouse monoclonal Ab (lane 1 and 6), as described in Methods. Click here for file
