# Supplementary figures and images for: Development of an ELISA for sensitive and specific detection of IgA autoantibodies against BP180 in pemphigoid diseases
Source: Orphanet J Rare Dis. 2011 May 28;6:31. doi: 10.1186/1750-1172-6-31 (PMC3126693; doi:10.1186/1750-1172-6-31)

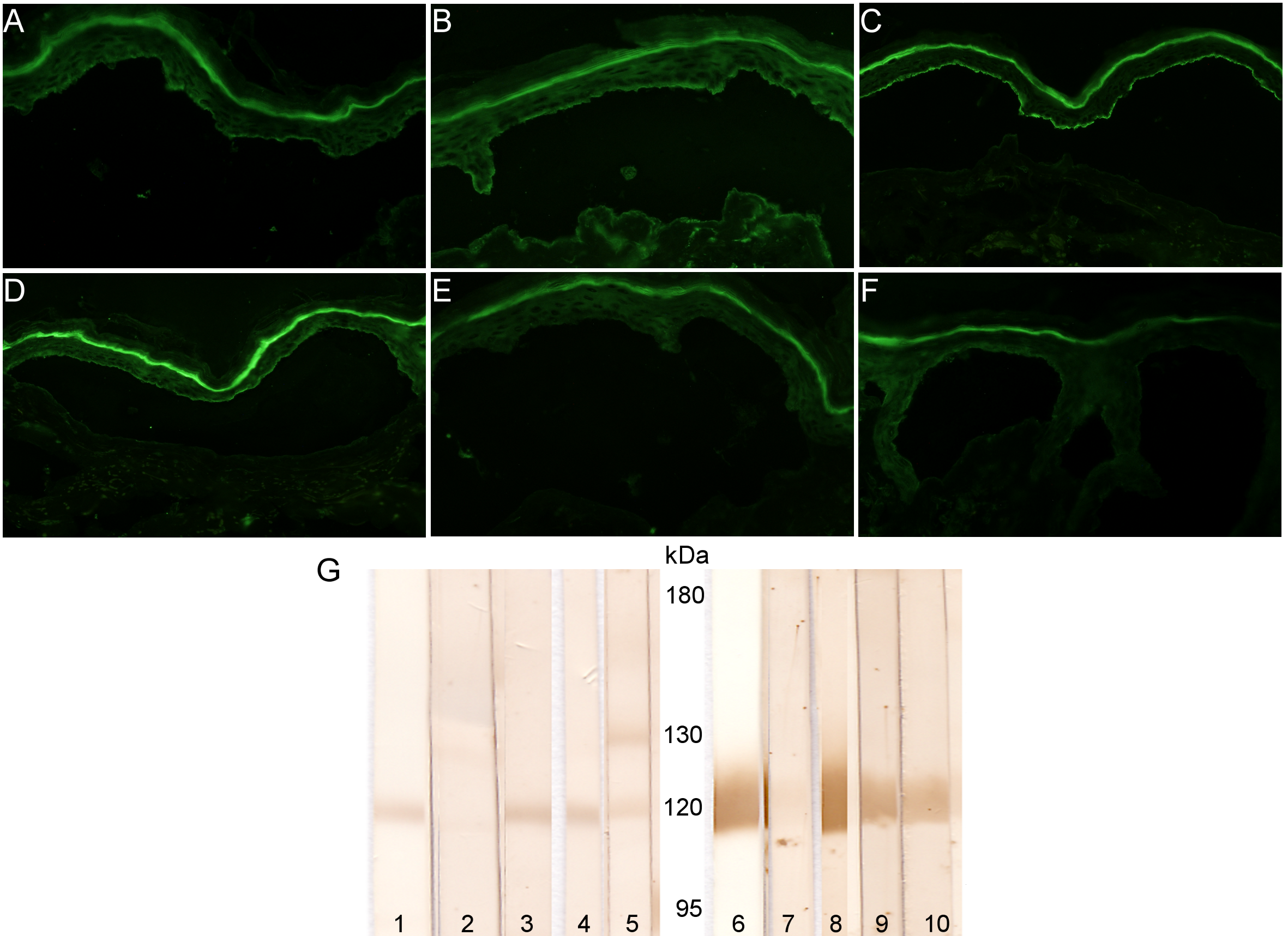

Supplement: Additional file 1 — Characterization of IgA autoreactivity in bullous pemphigoid (BP) patients. A-F: Cryosections of human salt split skin were incubated with BP sera (A, B, D, E), serum from a patient with IgA pemphigoid (C), and a healthy donor (F). IgA autoantibodies were detected using a FITC-labeled goat anti-human IgA antibody (magnification, 200x). G: Keratinocyte-derived shed (lanes 1-5) and recombinant BP180 ectodomain (lanes 6-10) were separated by 6% SDS-PAGE and electrophoretically transferred to nitrocellulose. The membranes were immunoblotted with serum from BP patients (lanes 3-5 and 8-10), a healthy donor (lane 2 and 7) and a BP180-specific mouse monoclonal Ab (lane 1 and 6), as described in Methods. [file 1750-1172-6-31-S1.TIFF]
